# AhR Ligands Differentially Regulate miRNA-132 Which Targets HMGB1 and to Control the Differentiation of Tregs and Th-17 Cells During Delayed-Type Hypersensitivity Response

**DOI:** 10.3389/fimmu.2021.635903

**Published:** 2021-02-19

**Authors:** Osama A. Abdulla, Wurood Neamah, Muthanna Sultan, Saurabh Chatterjee, Narendra Singh, Mitzi Nagarkatti, Prakash Nagarkatti

**Affiliations:** ^1^Department of Pathology, Microbiology and Immunology, School of Medicine, University of South Carolina, Columbia, SC, United States; ^2^Environmental Health and Disease Laboratory, Department of Environmental Health Sciences, Arnold School of Public Health, University of South Carolina, Columbia, SC, United States

**Keywords:** AhR, miR-132, HMGB1, Foxp3, IL17

## Abstract

Aryl hydrocarbon receptor (AhR), is a transcription factor and an environmental sensor that has been shown to regulate T cell differentiation. Interestingly, AhR ligands exert varying effects from suppression to exacerbation of inflammation through induction of Tregs and Th-17 cells, respectively. In the current study, we investigated whether the differential effects of AhR ligands on T cell differentiation are mediated by miRNA during delayed-type hypersensitivity (DTH) reaction against methylated Bovine Serum Albumin (mBSA). Treatment of C57BL/6 mice with TCDD attenuated mBSA-mediated DTH response, induced Tregs, decreased Th-17 cells, and caused upregulation of miRNA-132. TCDD caused an increase in several Treg subsets including inducible peripheral, natural thymic, and Th3 cells. Also, TCDD increased TGF-β and Foxp3 expression. In contrast, treating mice with FICZ exacerbated the DTH response, induced inflammatory Th17 cells, induced IL-17, and RORγ. Analysis of miRNA profiles from draining lymph nodes showed that miR-132 was upregulated in the TCDD group and downregulated in the FICZ group. Transfection studies revealed that miRNA-132 targeted High Mobility Group Box 1 (HMGB1). Downregulation of HMGB1 caused an increase in FoxP3+ Treg differentiation and suppression of Th-17 cells while upregulation of HMGB1 caused opposite effects. Moreover, TCDD was less effective in suppressing DTH response and induction of Tregs in mice that were deficient in miR-132. In summary, this study demonstrates that TCDD and FICZ have divergent effects on DTH response and T cell differentiation, which is mediated through, at least in part, regulation of miRNA-132 that targets HMGB1.

## Introduction

Aryl hydrocarbon receptor (AhR), a part of the family of basic helix-loop-helix transcription factors, responsible for regulating the toxicity induced by 2, 3, 7, 8-Tetrachlorodibenzo-p-dioxin (TCDD) (1). AhR is located in the cytosol of the cells as an inactive complex with numerous proteins such as chaperone protein hsp 90 (a 90 KD heat shock protein). Upon activation by its ligands, AhR translocates to the nucleus and undergoes conformational changes, and binds to its heterodimeric partner the aryl hydrocarbon receptor nuclear translocator (ARNT). The AhR-ARNT complex then binds to DNA recognition regions that contain xenobiotic response elements (XREs) or dioxin response elements (DRES) located in the promoter of AhR-responsive genes leading to regulation of their expression (2–4).

In addition to xenobiotic metabolism, studies have shown that AhR activation also plays a critical role in the regulation of the immune system (5, 6), specifically the differentiation of T cells into regulatory T cells (Tregs) and Th17 cells (7). Over the past several years, researchers have discovered a wide range of AhR ligands, both natural and synthetic (2, 3). The identification of these ligands has increased the knowledge of the physiological functions of AhR (8). TCDD, commonly known as dioxin, is an environmental pollutant and halogenated aromatic hydrocarbon, which is well-characterized for its toxic effects on the immune system (2). Because of its high binding affinity with AhR, TCDD is considered as the prototype for studying the bioactivity of the AhR. Today, TCDD is the AhR ligand of choice for studies meant at understanding the mechanisms by which the AhR activation regulates immune functions (9). In recent studies, TCDD has been shown to induce functional regulatory T cells that express Foxp3 (10). Several studies have also examined the use of TCDD on AhR activation in the treatment of autoimmune diseases (2, 10, 11), such as experimental colitis, multiple sclerosis, and animal models for type I diabetes. FICZ (6-formylindolo [3,2-b] carbazole), another AhR ligand, is a tryptophan-derived endogenous compound. It is a UV-induced photoproduct created in the skin that also binds AhR with very high affinity (11, 12). Studies have shown that FICZ promotes primarily the differentiation of Th-17 cells (13). Moreover, when FICZ was combined with the antigenic emulsion used to induce EAE, FICZ significantly boosted EAE development in wild- type (C57BL/6) mice but not in AhR-deficient (AhR knockout) mice (10, 14). In another study, it was reported that FICZ treatment worsened colitis in mice and there was a significant increase in Th17 cells in the mice (2). The mechanisms through which AhR ligands trigger pro-inflammatory vs. anti-inflammatory T cells remain unclear.

MicroRNA (miRNA) are a class of non-coding RNAs comprising of ~22 nucleotides that regulate the expression of a large set of genes at posttranscriptional levels (15). miRNA regulate their target mRNAs either by silencing or cleavage of mRNA (16, 17). It has been shown that animal miRNA identify their targets using a small nucleotide sequence known as seed sequence located at the 5′-end of target mRNA (18). It has been documented that a given mRNA is controlled by several miRNAs and also a given miRNA has many targets mRNAs (19, 20). AhR activation has been shown to regulate miRNA expression leading to the control of inflammatory responses (21).

In this study, we investigated how different AhR ligands (TCDD and FICZ) can impact the delayed-type hypersensitivity (DTH) reaction induced by mBSA, which is known to trigger Th17 cells (22). We also investigated if these ligands would induce differential levels of miRNA which in turn many impact the differentiation of inflammatory Th-17 and anti-inflammatory Tregs. Our studies demonstrated that while TCDD attenuated the DTH response, FICZ exacerbated the response. TCDD induced Tregs while FICZ induced Th-17 cells. We identified miR-132 which was upregulated by TCDD while being downregulated by FICZ. Lastly, using transfection experiments and the use of mice deficient in miR-132, we demonstrated that miR-132 targets HMGB1 which in turn regulates Treg induction. The current study demonstrates that the differential induction of Tregs vs. Th17 cells by AhR ligands may depend on the miRNA induced by these ligands. Our study also identifies HMGB1 as a unique molecule targeted by miRNA following AhR activation that regulates inflammation.

## Materials and Methods

### Animals

Female C57BL/6 (BL6) mice, at an average weight of 20 g and aged 8–10 weeks, were purchased from Jackson Laboratories (Bar Harbor, ME). Male and female mice exhibit differential susceptibilities to TCDD-AhR mediated transcriptomic response (23). It is for this reason that we did not mix male and female mice. We have provided this explanation in the text. We received miR-132/212 knockout mice as a breeding pairs from Dr. Mary Cheng, University of Toronto Mississauga, Mississauga, ON, CA as a gift. Mice were maintained and housed in a light- and temperature-controlled facility and allowed ad libitum access to water and diet at the University of South Carolina School of Medicine, Animal Research Facility. All experiments were approved by Institutional Animal Care and Use Committee animal protocol.

### Chemicals and Other Reagents

TCDD was kindly provided by Dr. Steve Safe (Institute of Biosciences & Technology, Texas A&M Health Sciences Center, College Station, Texas). FICZ was purchased from Enzo Life Sciences (Farmingdale, NY). Both TCDD and FICZ dissolved in DMSO were used for *in vitro* studies and dissolved in corn oil used for *in vivo* studies. mBSA and corn oil were purchased from Sigma-Aldrich (St. Louis, MO). RPMI 1640, L-Glutamine, penicillin-streptomycin, HEPES, PBS, and FBS were purchased from Invitrogen Life Technologies (Carlsbad, CA). AhR antagonist (CH223191) was purchased from Sigma-Aldrich, N.C. Fluorophore labeled monoclonal antibodies (mAbs) such as CD3-Alexa 488, CD4-PE/Cy7, CD8-Alexa 700, Foxp3-APC, IL-17-FITC, TGF-β1- PerCP-Cy5.5, IL10-PE, and Helois- PE–Dazzle, used for the flow cytometry, were purchased from Bio Legend (San Diego, CA) and Thermo fisher (Grand Island, NY). For intracellular staining, we used fixation/permeabilization kits from BD Biosciences for IL-17, and Foxp3 fix/perm buffer from Thermo fisher (Grand Island, NY).

### Induction of DTH in C57BL/6 Mice and Treatment With AhR Ligands (TCDD and FICZ)

To induce DTH in mice (C57BL/6), mBSA was first used to sensitize mice. All the sensitized mice were then rechallenged with mBSA in footpads as described previously (24–26). In brief, C57BL/6 mice (*n* = 5 mice per experimental group) were first sensitized with (1.5 mg/ml) mBSA (Sigma-Aldrich, MO) emulsified in complete Freund's adjuvant (CFA, Sigma Aldrich) by subcutaneous injection (100 μl/hind flank). All sensitized mice were then divided into four groups: Control (PBS), Vehicle (corn oil), TCDD (10 μg/kg body weight), and FICZ (50 μg/kg body weight). On day 5, each group of mice was treated according to treatment regimens by intraperitoneal (i.p.) injections as described previously (2, 27). On day 6, mice from all the four groups were rechallenged by intradermal injection of 10 mg/mL mBSA in PBS (20 μl/footpad) into both footpads. Each group contained at least five mice and the experiments were repeated at least three times. The footpad thickness of all groups of mice was measured using an engineer's calipers 48 h. after secondary challenge. The footpad swellings were calculated in percentage using the following formula: [(thickness (mBSA rechallenged footpad)—the thickness (PBS rechallenged footpad))/thickness (PBS rechallenged footpad)]/100.

### Histological Evaluation of the Footpads

Forty-eight hours after the rechallenge of footpads the mice were euthanized and footpads distal to the ankle were cut and preserved in Cal-Rite (Richard-Allan Scientific, Kalamazoo, MI) for at least 21 days to decalcify the footpad. The footpads were then sectioned into 5-μm thick sections and stained using hematoxylin and eosin (H&E). Infiltration of the immune cells in the footpad section of various treated groups was examined and analyzed by the Cytation 5 microscopic system (BioTek) as described previously (26, 27).

### Flow Cytometry Analysis to Analyze Cell Profiles in Draining Lymph Nodes (DLNs)

To determine the effect of various AhR ligands on immune cells in mice with DTH, draining lymph nodes (DLN: popliteal and inguinal lymph node) were harvested on day 2 post-secondary challenge and treatments. In brief, single-cell suspensions of harvested lymph nodes were first prepared, and then the cells were cultured overnight in a 6-well plate. The following day, the cells were incubated in the presence of Leukocyte Activation Cocktail from BD Biosciences (BD Biosciences, San Diego, CA) for four hrs. The cells were then collected, washed twice, and then stained using anti-mouse fluorophore-labeled mAbs. The cells (1 × 10^6^ cells) were incubated together with Fc Blocker reagent, which purchased from BD Biosciences (San Diego) for 10 min and then conjugated antibodies (FITC –conjugated anti-CD3, BV785-conjugated anti–CD45, APC/cy7-conjugated anti-CD4 and Alexa Fluor 700-conjugated anti-CD8 which purchased from BD Biosciences (San Diego) for 20–30 min at 4°C. After incubation, the cells were washed twice with FACS buffer (1 × PBS containing 2% fetal bovine serum). The stained cells were then analyzed using a flow cytometer (BD FACSCelesta) and the data were interpreted using FlowJo v10 software. Intracellular Cytokine Staining Kit (BD biosciences) was used for IL17a and Foxp3 using fix/perm buffer from (Thermo fisher) and following the manufacturer's instructions. In brief, the cells were first stained for cell surface marker (CD4) and after washing, the cells were fixed using either IL17 or Foxp3 fixation/permeabilization buffer. The cells were washed with PBS and then stained using anti-mouse antibodies (APC-conjugated anti–Foxp3, FITC-conjugated anti–IL-17, PE-conjugated anti–IL-10, PerCP-Cy5.5–conjugated anti–TGF-β, and PE–Dazzle–conjugated anti-Helios), which purchased from BD Biosciences (San Diego). The stained cells were analyzed by a flow cytometer (BD FACSCelesta). Data were analyzed with FlowJo v10 software.

### Assessment of Cytokines in Cell Culture Supernatants

To assess cytokines in cell culture supernatants, an enzyme-linked immunosorbent assay (ELISA) was performed. In brief, the cells were isolated from draining lymph nodes of mice with DTH and treated with vehicle, TCDD or FICZ. The cells (1 × 10^6^ cells/well) were then plated in a 96-well plate and cultured in complete RPMI medium overnight for spontaneous cytokine secretion. The following day, the cell supernatants were collected and either used immediately or stored at −20°C. ELISA kits for TGF-β and IL-17 cytokines were obtained from BioLegend (BioLegend, San Diego, CA). Following the protocol of the company, EILSA was performed using supernatants from cultured cells. Absorbance was measured at 450 nm by using a Victor (2) 1420 counter (Wallac) as described previously (26).

### Real-Time Quantitative PCR (RT-qPCR) to Determine the Expression of FoxP3, IL-17, IL-10, TGF-β, RORγT, and HMGB1 in DLN

To determine the expression of FoxP3, IL-17, IL-10, TGF-β, RORγT, and HMGB1 in DLN of the four groups, we performed RT-qPCR. In brief, cDNAs were generated using total RNAs isolated from the cells harvested from DLN of the four groups of mice. SSO Advanced™ SYBR green PCR kit from Bio-Rad (Hercules, CA, USA) was used. The following primers (Primer Bank, Harvard Medical School) were used for RT-qPCR (Table 1).

**Table 1:** Primer sequences for RT-qPCR.

**Gene**	**Primers**	**Sequence (5′-3′)**
Foxp3	Forward Reverse	CCCATCCCCAGGAGTCTTG ACCATGACTAGGGGCACTGTA
IL-17	Forward Reverse	CTCCTGCTTCTAGGCTGGTTG CCACCTGGCACTTCGAGTTAG
TGF-β	Forward Reverse	CGCTGCCCTTAAAAATATGGC GAGCCCCCTTTGTCTGAACTG
IL-10	Forward Reverse	CCCATTCCTCGTCACGATCTC TCAGACTGGTTTGGGATAGGTTT
HMGB1	Forward Reverse	GCCAGGAGAGCACAAGACAA GCAACGACACCAATGGATAAACC
RORγT	Forward Reverse	CAAGTTTGGCCGAATGTCC CTATAGATGCTGTCTCTGC
β -actin	Forward Reverse	GGCTGTATTCCCCTCCATCG CCAGTTGGTAACAATGCCATGT

RT-qPCR was performed using the following PCR cycles (40 cycles) and under these conditions: initial activation step (15 min at 95°C), denaturing temperature (15 s at 94°C), annealing temperature (30 s at 60°C), and extension temperature and fluorescence data collection (30 s at 70°C) were used. Using NE 14 2_DDCt, where Ct is the threshold cycle to detect fluorescence, normalized expression (NE) of mRNAs was calculated, and fold change of mRNA levels was normalized to β-actin (a housekeeping gene, *ACTB*). The Student's *t*-test was performed using GraphPad version 6.01 (GraphPad Software, INC., San Diego, CA) to determine significant differences in mRNAs level in the draining lymph nodes of all the four groups. Differences between treatment groups were considered significant when *p* < 0.05.

### miRNA Arrays to Evaluate miRNA Profile in DLN Post-treatment of DTH Mice

The cells from DLNs from mice with DTH and treated with VEH or TCDD or FICZ were first isolated and after washing thoroughly, the cells were suspended in TRIzol reagent from a miRNeasy mini-kit. Total RNAs including microRNAs were isolated using miRNeasy mini-kit and following the manufacturer's instructions (QIAGEN, Valencia, CA). miRNAs arrays were performed by Johns Hopkins sequencing core facility, Baltimore. The data generated from the miRNAs arrays were checked and verified using quality control and normalization was performed as described earlier (28). miR arrays data were submitted to Array Express (Accession number: E-MTAB-10117; Available online at: http://www.ebi.ac.uk/arrayexpress/submit/overview.html). Linear fold-changes in miRNA down-regulation or up-regulation were estimated to compare the differences of all the miRNAs expressed between TCDD, FICZ or vehicle group. A linear fold-change of at least (1.5/−1.5) was considered as a cut off value for the inclusion of a miRNA with a change. Determination of miRNAs interaction and pathway analysis was done using Ingenuity Pathway Analysis (IPA; Qiagen; www.ingenuity.com).

### Studies Using AhR Antagonist on TCDD and FICZ-Mediated Alterations in DTH Response

To determine whether TCDD- or FICZ-induced effects *in vivo* were mediated through activation of AhR, we used AhR-specific antagonist CH223191 (CH). DTH was inducted as described above and AhR antagonist CH (10 mg/kg body weight) was injected i.p. 2 h prior Veh, TCDD or FICZ treatment. The mice were observed for DTH response and then euthanized 72 h after the rechallenge with mBSA. DLN were harvested and single cells were prepared and analyzed for various markers.

### RT-qPCR to Validate the Expression of miR-132-3p and Its Target Gene *HMGB1* in DLNs

After analyzing miRNAs profiles of draining lymph node cells, up-or down-regulated miRNAs were identified in mBSA+TCDD and mBSA+FICZ groups in comparison to the vehicle group. Next, we performed RT-qPCR to validate the expression of miR-132-3p (5′UAACAGUCUACAGCCAUG GUCG) and its target gene *HMGB1* in lymph node cells obtained from vehicle- or TCDD- or FICZ-treated groups. In brief, total RNAs including miRNAs from draining lymph nodes of various treated groups as described earlier were isolated using miRNeasy kit from Qiagen and following the manufacturer's instructions (QIAGEN, Valencia, CA). miScript primer assays kit and miScript SYBR Green PCR kit from QIAGEN were used and RT-qPCRs were performed following the protocol of the company (QIAGEN, Valencia, CA). For RT-qPCR, 40 cycles using the following conditions: 15 min at 95°C (initial activation step), 15 s at 94°C (denaturing temperature), 30 s at 60°C (annealing temperature), and 30s at 70°C (extension temperature and fluorescence data collection) were used. Normalized expression (NE) of miRNAs was calculated using NE 14 2_DDCt, where Ct is the threshold cycle to detect fluorescence. The data were normalized to miRNAs against internal control miRNA (SNORD96A) and fold change of miRNAs was calculated against control miRNA (SNORD96A) and treatment groups: mBSA+TCDD and mBSA+FICZ were compared with mBSA+Vehicle group. To define significant differences in miRNAs level in the lymph node cells of mBSA+TCDD or mBSA+FICZ or mBSA+Vehicle groups, Student's *t*-tests were performed using GraphPad version 6.01 (GraphPad Software, INC., San Diego, CA). Differences between treatment groups were considered significant when *p* < 0.05. To determine the expression of the *HMGB1* gene, mRNA levels were normalized to β-Actin.

### Transfection of Draining Lymph Node Cells With miR-132-3p

The transfections of draining lymph node cells were performed as described earlier (29, 30). In brief, cells isolated from draining LNs from mBSA-immunized mice were either transfected with scrambled miRNAs (Mock) or with mature miR-132-3p mimic (5′UAACAGUCUACAGCCA UGGUCG) or miR-132-3p (5′UAACAGUCUACAGCCAUGGUCG) inhibitor using the HiPerFect transfection kit (Qiagen) and following the company's protocol. The draining lymph node cells (4 × 10^5^ cells/well) were first cultured in complete RPMI medium in a 24-well plate, The following day, the cells were transfected with mock, mimic or inhibitor or both mimic and inhibitor. After transfection, the cells were incubated at 37°C for 24 h. Next, the transfected cells were collected and total RNAs including miRNAs were isolated using miRNeasy kit as described above (Qiagen). cDNAs were synthesized using total RNAs and miScript II RT kit (Qiagen) for target genes expression analysis and miRNAs validation.

### Statistical Analysis

Statistical analyses were pursued using GraphPad Prism software (GraphPad Software version 6). The data is shown as mean ± S.E.M in groups of 5 mice. A one-way ANOVA test was used to compare three or more groups. Student's *t*-test was used to compare data between two groups. Statistically significant differences were depicted in figures as ^*^p < 0.05, ^**^p < 0.01, ^***^p < 0.001, ^****^p < 0.0001.

## Results

### AhR Ligands, TCDD and FICZ, Have Opposite Effects on mBSA-Induced DTH in Mice

**Figure 1:**
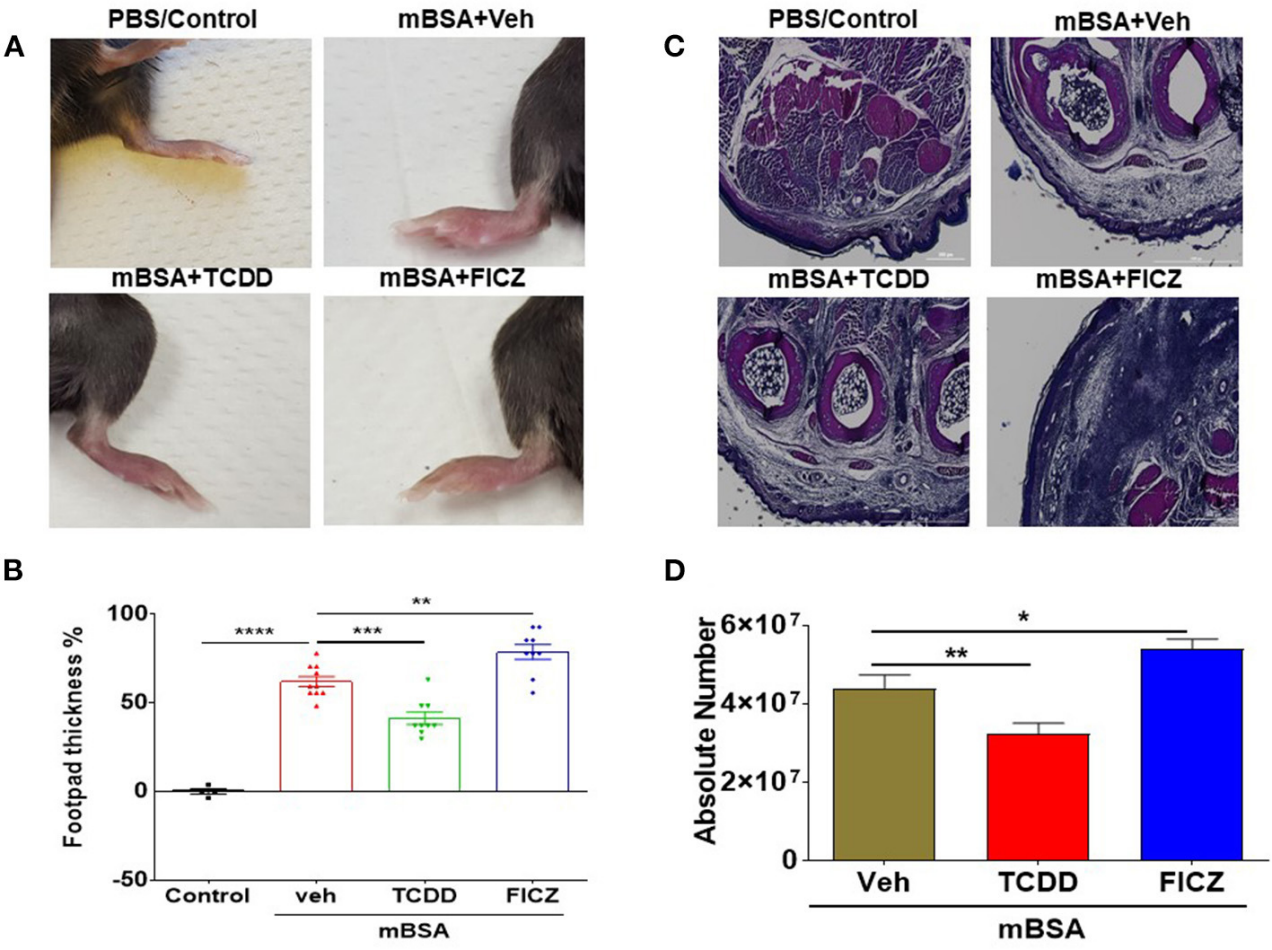
AhR ligands (TCDD and FICZ) have opposite effects on mBSA-induced DTH in mice. DTH was induced by injecting mBSA and these mice were treated with vehicle, TCDD or FICZ, as detailed in Methods. **(A)** Shows footpad thickness in mice with DTH. **(B)** Showing the percentage change in footpad thickness in all the groups of mice. **(C)** H&E (4X magnification) staining of histological sections of footpads from all the four groups of mice. **(D)** Absolute cell number of cells in draining lymph nodes of various groups. Vertical Bars represent Mean ± SEM and significant difference in total cell numbers are shown with asterisks (^*^p < 0.05, ^**^p < 0.01, ^***^p < 0.001, ^****^p < 0.0001) based on one-way ANOVA.

As described in Materials and Methods, mBSA was used to induce DTH in C57BL/6 mice. There were four groups of mice: Vehicle Control, mBSA+Vehicle, mBSA+TCDD, and mBSA+FICZ. Each group had 5 mice and the experiments were repeated at least three times. Data obtained from this study showed that mBSA+Veh treated mice showed significant DTH responses in the footpads of mice when compared to mice treated with vehicle alone (Figures 1A,B). Treatment of mBSA-injected mice with TCDD caused significant suppression of DTH responses when compared to mBSA+vehicle-treated mice (Figures 1A,B). However, FICZ treatment caused a significant increase in DTH in mice, when compared to vehicle-treated mice (Figures 1A,B). Upon histological analysis of the footpads, there was an increase in infiltrating cells in mBSA+Vehicle mice, when compared to vehicle-treated control mice (Figure 1C). However, TCDD treatment reduced the number of infiltrating cells, when compared to the mBSA+Vehicle group (Figure 1C). In contrast, there was a further increase in the number of infiltrating cells in FICZ-treated mice, when compared to the DTH+Vehicle group (Figure 1C). Furthermore, upon examination of the cellularity of draining lymph nodes, there was also a significant increase in the total number of cells in the mBSA+Vehicle group, when compared to PBS control. TCDD treatment, however, significantly reduced the total number of cells in the draining lymph nodes (DLNs), when compared to the vehicle-treated group (Figure 1D). FICZ treatment had the opposite effects of TCDD. FICZ treatment significantly increased the number of cells in draining lymph nodes of mice, when compared to mBSA +Vehicle group (Figure 1D). Taken together, these data showed that AhR ligands, TCDD, and FICZ, had opposite effects on mBSA-induced DTH in mice (Figures 1A–D).

### TCDD Causes a Reduction in T Cells Whereas FICZ Increases the Number of T Cells in mBSA-Induced DTH in Mice

**Figure 2:**
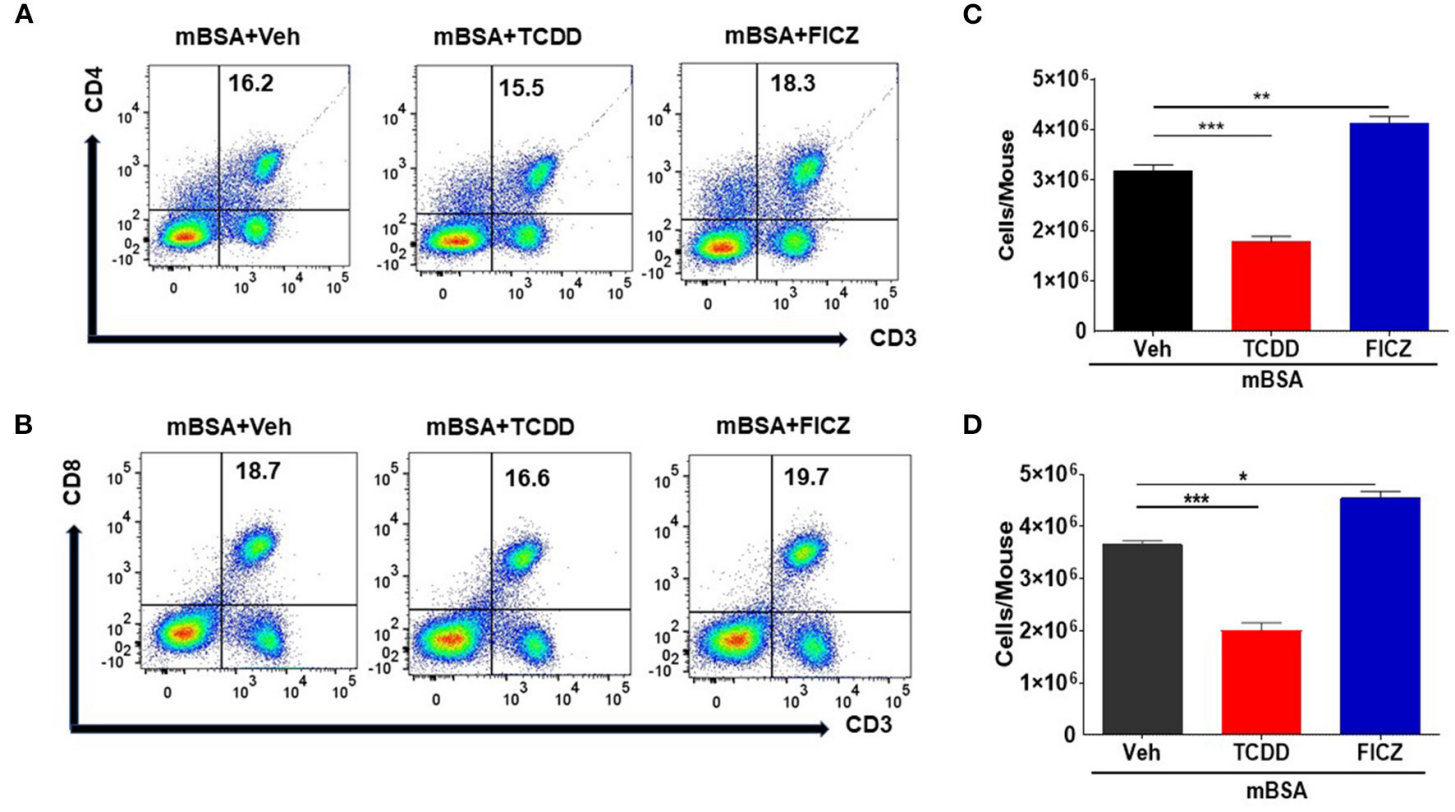
TCDD treatment reduces T cell infiltration in DLNs in mBSA-induced DTH. DTH was induced as described in Figure 1 legend and DLNs were analyzed for T cells. **(A)** Representative plots from FlowJo software analysis of flow cytometry data showing cells double-stained for CD3 and CD4. **(B)** Shows absolute cell numbers of CD3+CD4+ T cells in DLNs of various treatment groups. These numbers were calculated based on the absolute number of cells in DLN multiplied by the percentage values of CD3+CD4+ cells obtained by flow cytometry, divided by 100. **(C)** Representative plots from FlowJo software analysis of flow cytometry data showing cells double-stained for CD3 and CD8. **(D)** Absolute number of CD3+CD8+ cells. The statistically significant differences between the groups were calculated using one-way ANOVA. Vertical bars represent Mean ± SEM and significant differences between groups were indicated by asterisks (^*^p < 0.05, ^**^p < 0.01, ^***^p < 0.001).

To understand the effects of AhR ligands, TCDD and FICZ, on mBSA-induced inflammation-causing DTH in mice, we determined the total number of T cell subsets present in draining lymph nodes of mice with DTH, treated with vehicle, TCDD or FICZ. Figures 2A,B show a representative flow cytometric analysis of T cells and Figures 2C,D show the total number of such cells calculated per mouse based on the percentage and absolute numbers of cells found in the draining lymph nodes. As shown in Figure 2C, there was a significant decrease in CD3+CD4+ T cells in TCDD treated mice, when compared to vehicle-treated mice. In contrast, there was a significant increase in the total number of CD3+CD4+ T cells in FICZ-treated mice (Figure 2C). We observed similar trends in CD3+CD8+ cell numbers in TCDD-treated (significant reduction in CD3+CD8+ T cells) and a significant increase in CD3+CD8+ cell numbers in FICZ-treated mice with DTH, when compared to vehicle-treated mice (Figure 2D). Together, these data demonstrated that TCDD treatment reduced the CD4+ and CD8+ T cells in the draining lymph nodes whereas, FICZ treatment increased the number of T cells, when compared to Vehicle treated mice with DTH (Figures 2A–D).

### AhR Ligands, TCDD and FICZ, Differentially Regulate Tregs in Mice With DTH

**Figure 3:**
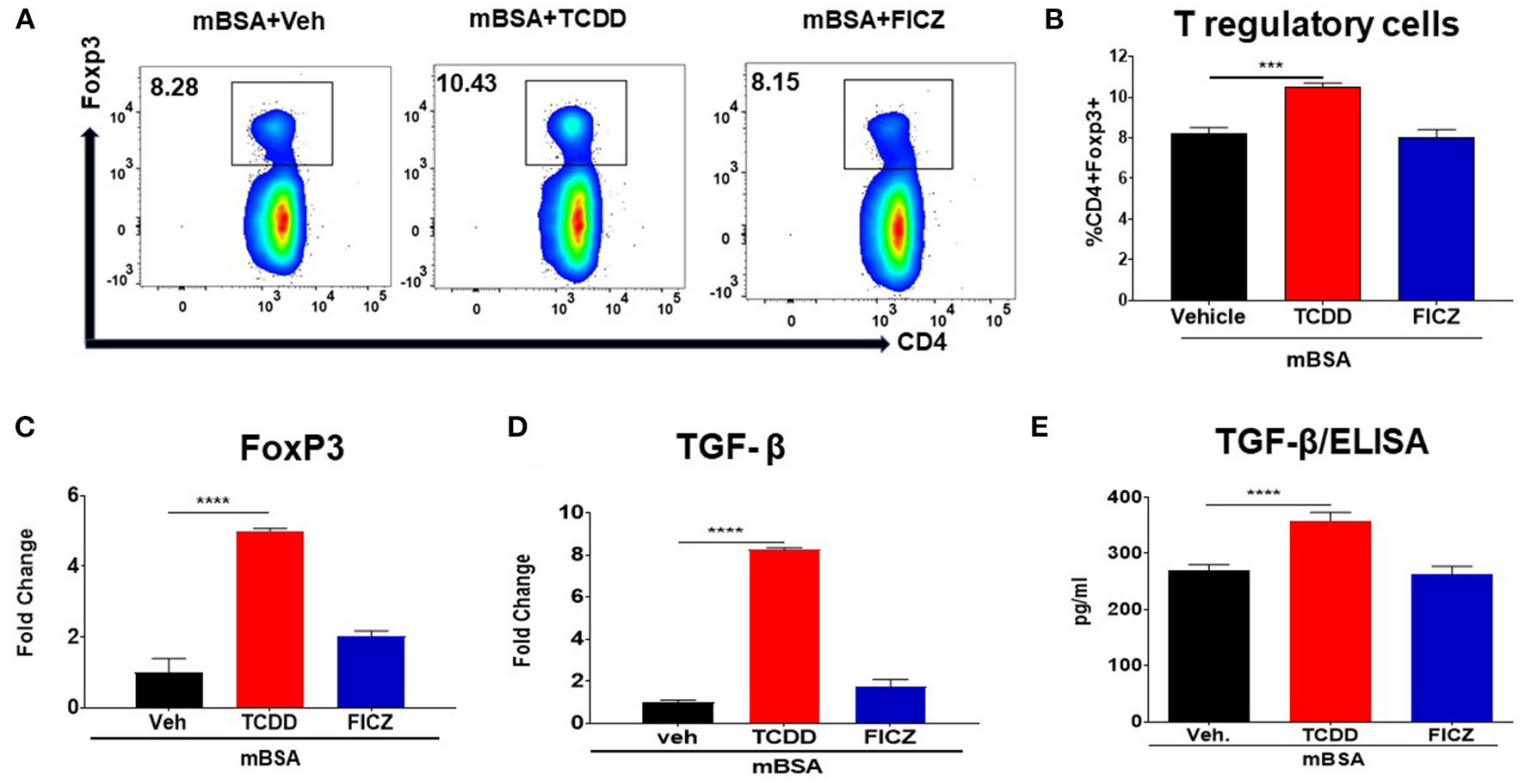
TCDD induces FoxP3+ Tregs during DTH response. DTH was induced as described in Figure 1 legend. Tregs, as well as expression of FoxP3 and TGF-β, were studied. **(A)** Representative plots from FlowJo software analysis of flow cytometry data showing CD4+ Foxp3+ cells from DLNs. **(B)** Percentage of CD4+Foxp3+ cells from groups of 5 mice. **(C)** qRT-PCR validation of mRNA expression of Foxp3 in DLN cells. **(D)** qRT-PCR based mRNA expression of TGF-β in DLN cells. **(E)** DLN cells were plated overnight in complete media and culture supernatants were harvested and subjected to ELISA for TGF-β. The statistically significant differences between the groups were calculated using one-way ANOVA. Vertical bars represent Mean ± SEM and significant differences between groups are shown with asterisks (^***^p < 0.001, ^****^p < 0.0001).

To better understand the differential effects of TCDD and FICZ, we investigated their effect on Tregs and Th17 cells in mBSA-induced DTH. Figure 3A shows a representative flow cytometric analysis and Figure 3B shows data from multiple mice tested in each group. TCDD increased the percentage of Tregs in draining lymph nodes of mice with DTH, when compared to vehicle controls. In contrast, FICZ treatment did not alter the percentage of Tregs (Figures 3A,B). Next, we evaluated the expression of Foxp3 (a marker for Tregs) and TGF-β (produced by Tregs) in draining LN cells obtained from three groups of mice (mBSA+Vehicle, mBSA+TCDD, and mBSA+FICZ). As shown in Figures 3C,D, there was a significant increase in FoxP3 and TGF-β expression in mBSA+TCDD group of mice, when compared to Vehicle-treated mice, while FICZ failed to cause an increase (Figures 3C,D). Upon evaluation of TGF-β secretion in supernatants of cultured LN cells by ELISA, a significant increase in TGF-β secretion was observed while FICZ failed to show such an increase (Figure 3E).

**Figure 4:**
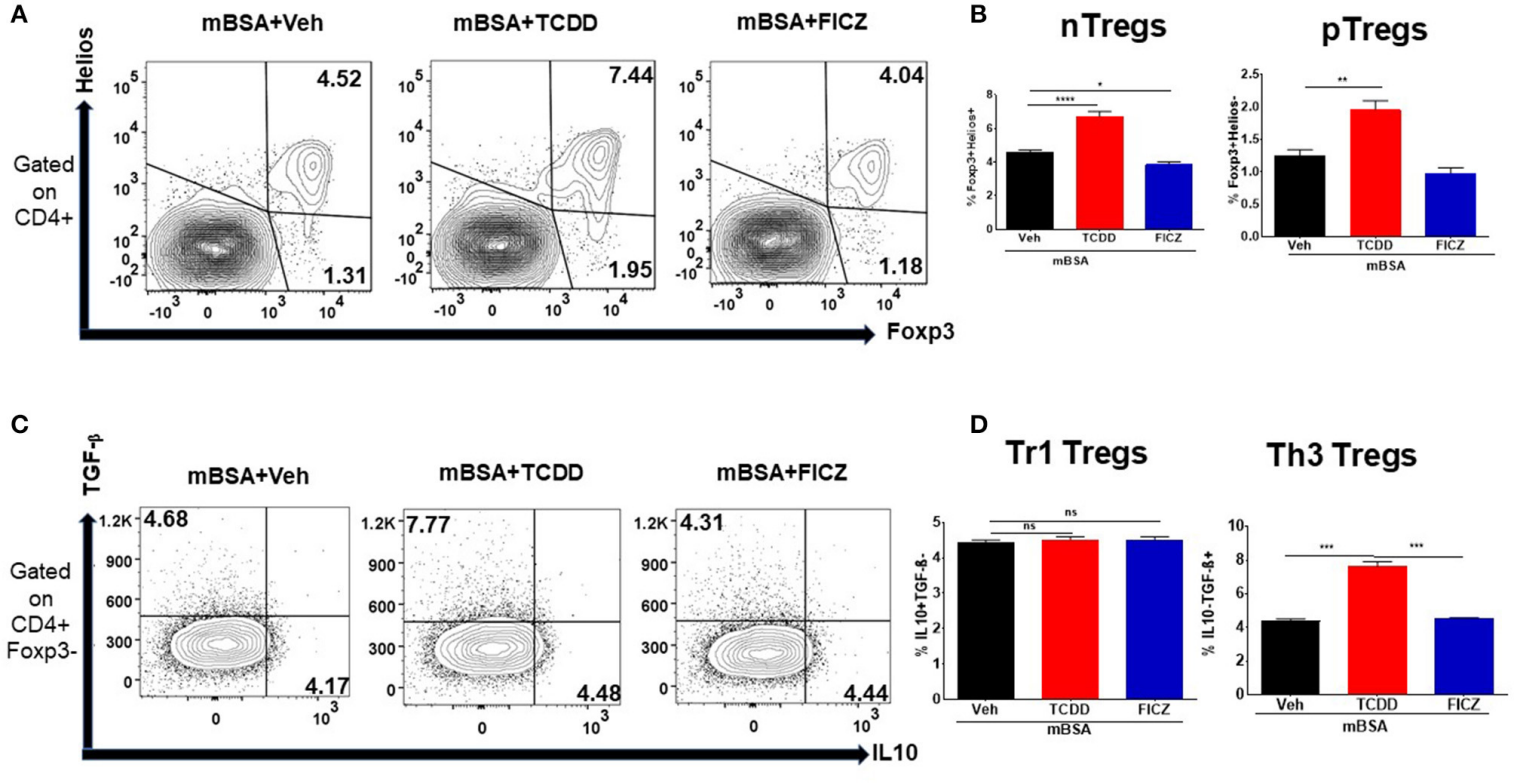
Effect of TCDD on Treg subsets. DTH was induced as described in Figure 1 legend and Treg subsets were studied using DLN cells and flow cytometric analysis for various markers. **(A)** Representative flow cytometry dot plots gated on CD4+ cells, displaying the percentage of natural Tregs (nTreg) present through co-expression of CD4, FoxP3, and Helios and the percentage of peripheral Tregs (pTreg) that are CD4+ and FoxP3+, and Helios-. **(B)** Percentage of (nTregs and pTregs) cells based on groups of 5 mice. **(C)** Representative flow cytometry dot plots gated on CD4+Foxp3- cells, displaying the percentage of Tr1 Tregs present through co-expression of CD4+Foxp3-IL-10+ and the percentage of Th3 Tregs that are CD4+Foxp3-TGF-β+. **(D)** Percentage of Tr1 and Th3 Tregs cells based on groups of 5 mice. Vertical bars represent Mean ± SEM and significant difference between groups were indicated by asterisks (^*^p < 0.05, ^**^p < 0.01, ^***^p < 0.001, ^****^p < 0.0001) based on one-way ANOVA.

We also examined the effect of TCDD and FICZ on Treg subsets in mice with DTH. To that end, DLN cells from mBSA injected mice treated with Vehicle or TCDD or FICZ were stained for various markers for thymic Tregs (nTregs) (CD4+/FoxP3+/Helios+), peripheral Tregs (pTregs) (CD4+/FoxP3+/Helios−), Th3 Tregs (CD4+/IL-10-/TGF-β+), and Tr1 Tregs (CD4+/IL-10+/TGF-β−). As shown in Figures 4A–D, there was a significant increase in the percentage of nTregs, pTregs and Th3 cells but not Tr1 cells in mBSA+TCDD group, when compared to mBSA +Vehicle group, while FICZ failed to cause an increase in any of these Treg subsets and in fact, decreased the percentage of nTregs (Figure 4). Taken together, these data demonstrated that TCDD increased the generation of all Treg subsets except Tr1 cells in DTH mice while FICZ failed to cause an increase in these Treg subsets.

### Effect of TCDD and FICZ on the Generation of Th17 Cells

**Figure 5:**
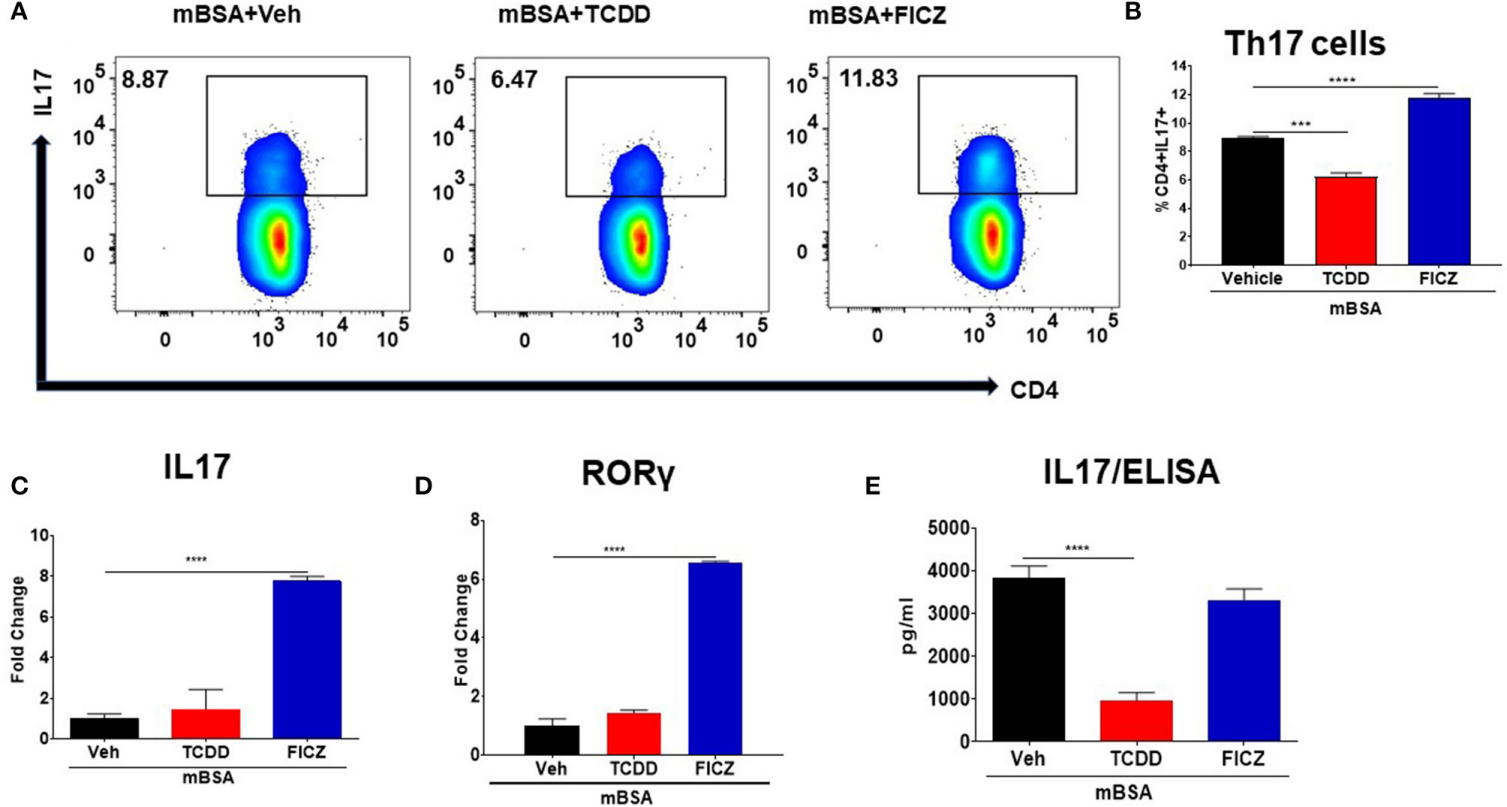
Effect of TCDD and FICZ on Th-17 cells during DTH response. DTH was induced as described in Figure 1 legend and DLNs were analyzed for Th-17 cells. **(A)** Representative plots from FlowJo software analysis of flow cytometry data showing CD4+IL7+ cells. **(B)** Percentage of CD4+IL17+ cells based on groups of 5 mice. **(C)** qRT-PCR validation of mRNA expression of IL17 in DLN cells. **(D)** qRT-PCR validation of mRNA expression of RORγ in DLN cells. **(E)** DLN cells were plated overnight in a complete medium and culture supernatants were harvested and subjected to ELISA for IL17. Vertical bars represent Mean ± SEM and significant differences between groups are shown with asterisks (^***^p < 0.001, ^****^p < 0.0001) based on one-way ANOVA.

Next, we examined the effect of TCDD and FICZ on the generation of Th17 cells in the DLNs of mice with DTH. The flow cytometry data showed that there was a significant increase in the percentage of Th17 cells in the draining lymph nodes of mBSA+FICZ mice when compared to mBSA+Vehicle mice (Figures 5A,B), while there was a significant decrease in Th17 cells percentage in mBSA+TCDD mice when compared to mBSA+Veh mice (Figures 5A,B). Upon evaluation of IL17 and RORγt expression in DLN cells, there was a significant increase in both IL17 and RORγt expression in the mBSA+FICZ group, when compared to the mBSA+Vehicle group (Figures 5C,D). TCDD treatment, on the other hand, had minimal or no effect on IL17 and RORγt expression (Figures 5C,D), when compared to the mBSA+Vehicle group. When the expression of IL17 was determined by ELISA, it was significantly decreased in LN cells treated with TCDD, when compared to Vehicle (Figure 5E). However, FICZ treatment had minimal or no effect on IL17 levels (Figure 5E). Data obtained from these studies demonstrated that AhR ligands, TCDD, and FICZ, have contrasting effects on the induction of Tregs and Th17 cells during DTH reaction.

### Analysis of miRNA Profile in Draining Lymph Nodes of Mice With DTH Treated With TCDD and FICZ

**Figure 6:**
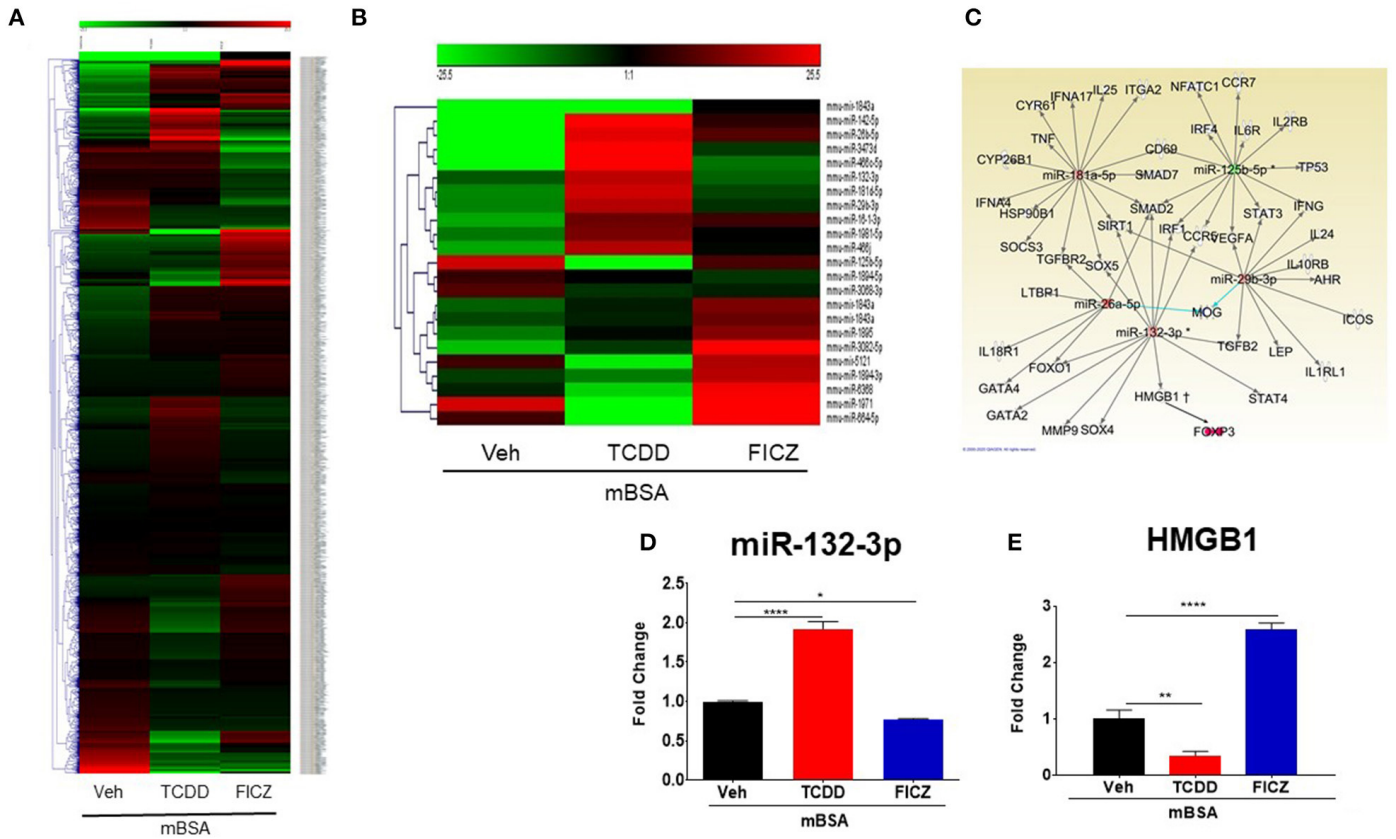
miRNAs expression profile from cells isolated from DLN of mice with DTH and treated with VEH, TCDD, and FICZ. **(A)** Heat map showing miRNAs expression profile in cells of DLN from mice with DTH treated with VEH, TCDD, and FICZ. The expression array (green to red) represents the scale of downregulated to upregulated expression array of miRNAs, respectively. **(B)** Heat map representing fold change (−1.5, +1.5 fold) expression profile of miRNAs that were significantly different between the groups. **(C)** IPA ingenuity software was used to establish interactions between miRNAs and the proinflammatory T cell pathway. **(D)** qRT-PCR validation of the expression of miR-132-3p in DLN cells. **(E)** qRT-PCR validation of mRNA expression of HMGB1 in DLN cells. Vertical bars represent Mean ± SEM and significant differences between groups are shown with asterisks (^*^p < 0.05, ^**^p < 0.01, ^****^p < 0.0001) based on one-way ANOVA.

Recent data have shown that miRNAs might regulate cytokines, pathway signaling, and inflammatory diseases (15, 31). To investigate the differences in the mode of action of the two AhR ligands (TCDD and FICZ), we performed miRNA arrays and analyzed the miRNAs profile in the DLN cells of mice with DTH treated with Vehicle or TCDD or FICZ. Cluster analysis of more than 3,000 miRNAs (Figure 6A) showed that TCDD and FICZ exhibited a different expression pattern of miRNAs than vehicle-treated mice. Several miRNAs were upregulated >1.5 in the TCDD group whereas, they were downregulated <−1.5 in the FICZ group (Figure 6B). Interestingly, there was only one microRNA, miR-132-3p, that showed potential regulation of Foxp3 indirectly by acting through its target, HMGB1 as seen from the analysis of altered miRNA using IPA (Figure 6C). This miRNA was upregulated in the mBSA+TCDD group but downregulated in the mBSA+FICZ group when compared to the mBSA+Vehicle group. We validated the expression of miR-132-3p and HMGB1 in DLN cells from the three groups by performing RT-qPCR. These data showed that miR-132-3p was significantly upregulated (Figure 6D) by TCDD but was downregulated by FICZ (Figure 6D), when compared to Vehicle (Figure 6D). Upon examination of HMGB1 expression, TCDD downregulated the expression of HMGB1 in DLN cells (Figure 6D) whereas, FICZ upregulated its expression (Figure 6E), when compared to the Vehicle group (Figure 6E).

**Figure 7:**
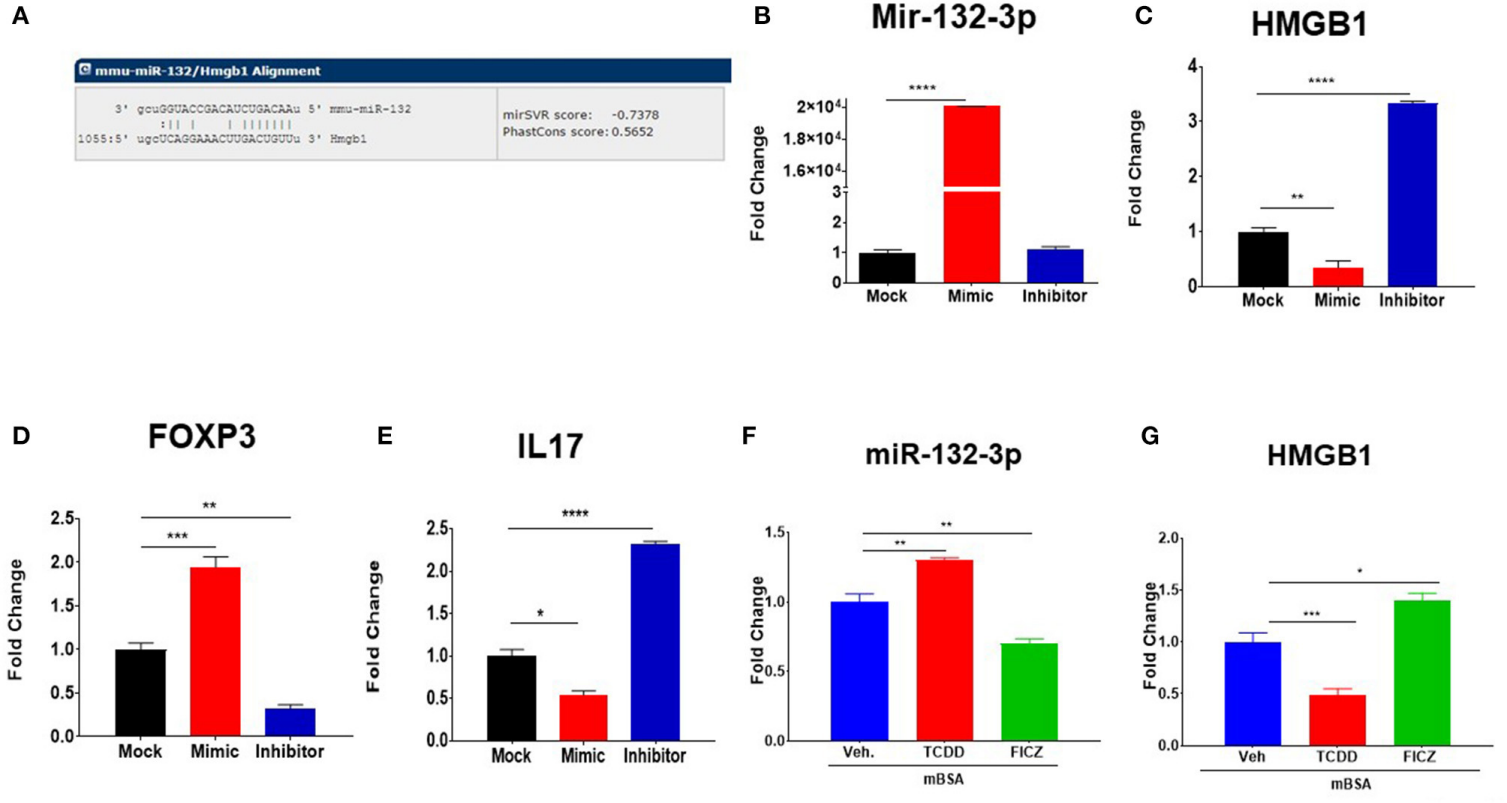
Transfection and alignment studies to investigate the targets of miR-132-3p. **(A)** Using sequence microrna.org miR-132-3p was highly predicted to target mRNA for HMGB1. DTH was induced as described in Figure 1 legend and DLN cells from mBSA-induced DTH mice were transfected with either scrambled miRNAs (Mock) or with mature miR-132-3p mimic or miR-132-3p as described in Methods. After transfection, the cells were incubated at 37°C for 24 h and analyzed for gene expression and miRNAs validation. **(B)** qRT-PCR based expression of miR-132-3p. **(C)** qRT-PCR-based expression of HMGB1. **(D)** qRT-PCR based expression of FoxP3. **(E)** qRT-PCR based expression of Il-17. **(F,G)** Naïve mice were treated with TCDD and FICZ and lymph node cells were tested using q-RT-PCR on the expression of miR-132-3p and HMGB1. Vertical bars represent Mean ± SEM and significant differences between groups are shown with asterisks (^*^p < 0.05, ^**^p < 0.01, ^***^p < 0.001, ^****^p < 0.0001) based on one-way ANOVA.

We studied *in silico* prediction analysis using microrna.org and found alignment of the miRNA-132-3p with the 3′UTR of the *HMGB1* target gene (Figure 7A). To further confirm the role of miR-132-3p in the regulation of HMGB1 expression, we performed transfection assays using miR-132-3p mimic and its inhibitor (Figure 7B). The expression of HMGB1 was significantly suppressed in the mimic group while it was significantly increased in the inhibitor group (Figure 7C). Additionally, FoxP3 expression was increased in the mimic group while it was decreased in the inhibitor group when compared to controls (Figure 7D). In contrast, IL-17 expression was decreased in the mimic group and increased in the inhibitor group (Figure 7E). These transfection experiments confirmed that miR-132-3p targets HMGB1.

To test if TCDD and FICZ-mediated effect on miRNA induction is seen only in mice with DTH, we injected these compounds into naïve mice and analyzed the lymph node cells 3 days later for miR-132-3p and HMGB1. The data showed that TCDD was able to induce miR-132-3p (Figure 7E) and suppress HMGB1 (Figure 7F) in naïve mice while FICZ caused opposite effects. These data suggested that the effect of TCDD and FICZ on miR-132-3p and HMGB1 was not restricted to immunized mice and could be seen even in naïve mice.

### Role of AhR in TCDD and FICZ-Mediated Alterations in the DTH Response and miRNA

**Figure 8:**
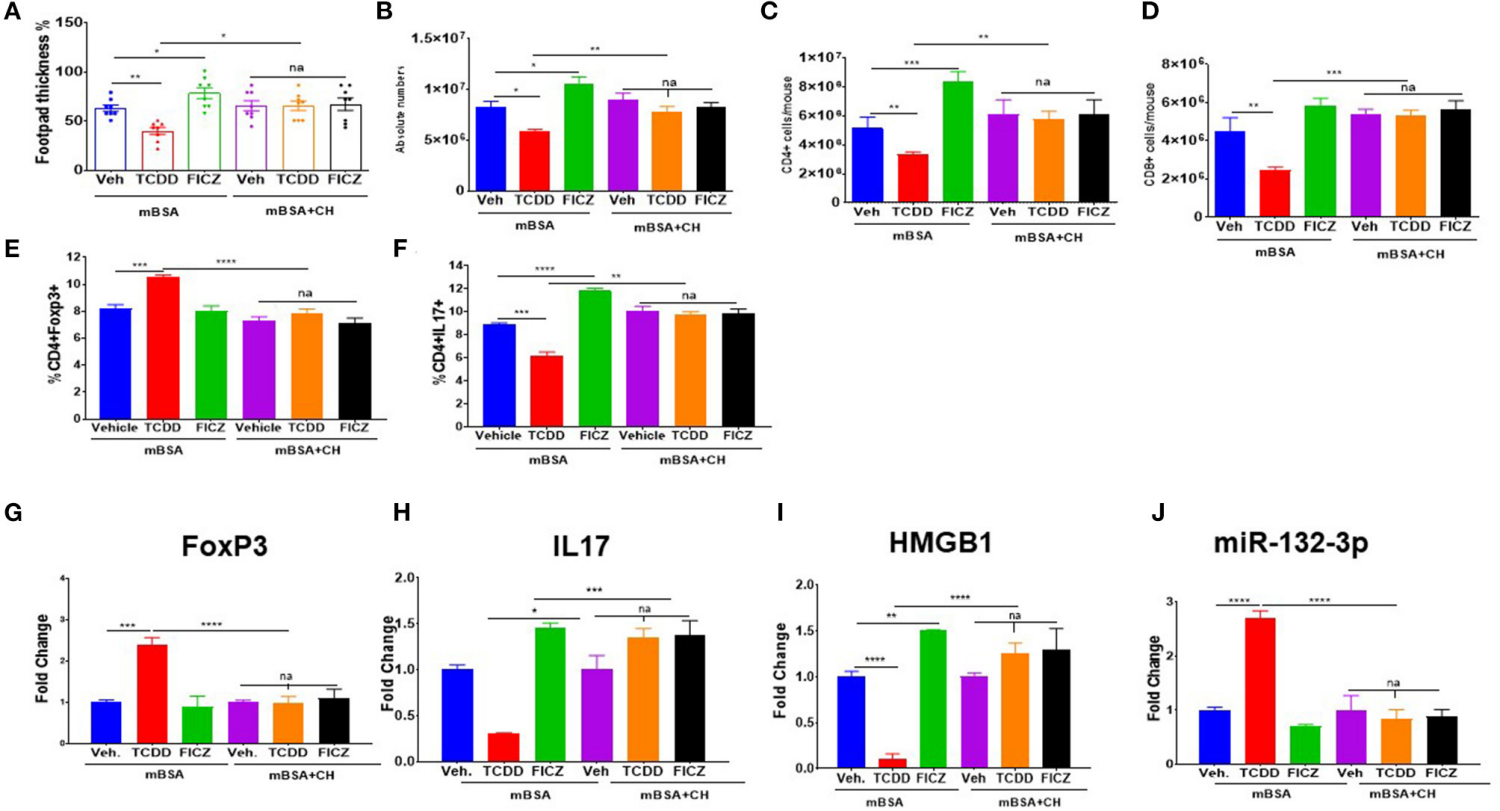
Effect of AhR antagonist (CH) on TCDD and FICZ-mediated alterations in the DTH response. DTH was induced by injecting mBSA and these mice were treated with vehicle, TCDD or FICZ. Additionally, these mice received AhR antagonist (CH) prior to treatment with FICZ and TCDD. The draining DLN were harvested and studied as follows: **(A)** Showing the percentage change in footpad thickness. **(B)** Absolute cell number in DLN. **(C)** Absolute cell number of CD3+CD4+ cells. **(D)** Absolute cell number of CD3+CD8+ T cells. **(E)** Percentage of CD4+FoxP3+ T cells. **(F)** Percentage of CD4+IL-17+ T cells. qTR- PCR-based expression of Foxp3 **(G)**, IL17 **(H)**, HMGB1 **(I)**, and miR-132-3p **(J)** in DLN cells. Vertical Bars represent Mean ± SEM using groups of 5 mice and significant differences between groups are shown with asterisks (^*^p < 0.05, ^**^p < 0.01, ^***^p < 0.001, ^****^p < 0.0001) based on one-way ANOVA.

To test whether the immunological changes mediated by TCDD and FICZ are regulated through activation of AhR, we used an AhR antagonist (CH) to investigate if the effects can be reversed. In this experiment, we used six groups of mice: mBSA+Veh, mBSA+TCDD, mBSA+FICZ, mBSA+CH+Veh, mBSA+CH+TCDD, and mBSA+CH+FICZ. The data showed that the ability of TCDD to suppress, and FICZ to enhance the DTH response as measured by footpad swelling, was reversed by CH (Figure 8A). Moreover, the effect of TCDD to suppress absolute cell numbers in the DLN, CD4+ T cells, CD8+ T cells, and Th17 cells while increasing FoxP3+CD4+ T cells was also reversed by CH (Figures 8B–F). Additionally, the ability of TCDD to induce FoxP3 and miR-132-3p, while suppressing the expression of IL-17 and HMGB1, was also reversed by CH (Figures 8G–J). As noted previously, FICZ caused opposite effects on all these markers when compared to TCDD, and furthermore, treatment with CH reversed the effects of FICZ. Together, these data suggested that TCDD and FICZ were acting through AhR to regulate the DTH response and various markers of inflammation.

### Use of miR-132 KO Mice to Study the Role of TCDD and FICZ in the Regulation of DTH and Treg vs. Th17 Differentiation

**Figure 9:**
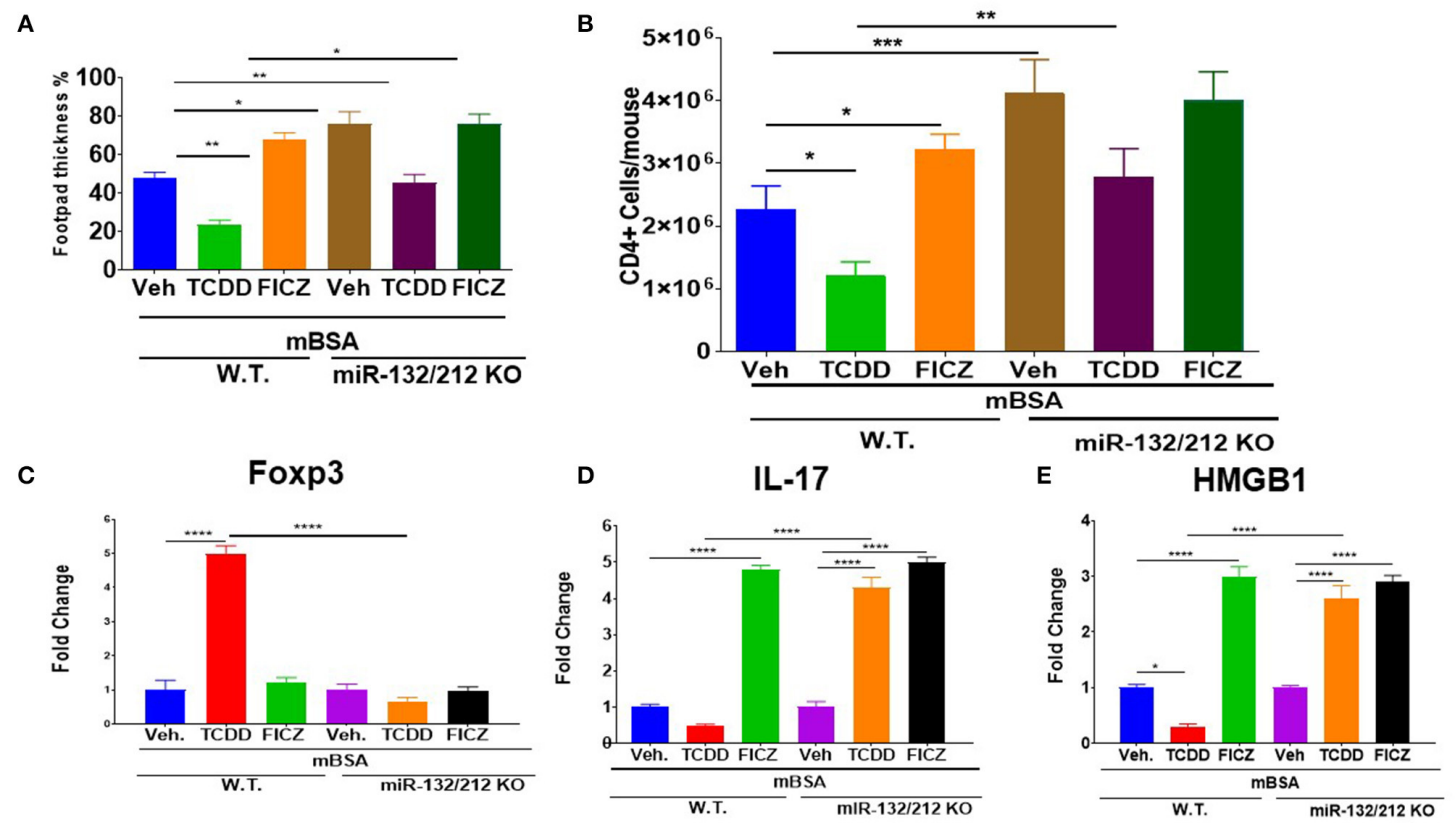
Studies using miR-132/212 KO mice to assess the role of TCDD and FICZ on DTH response. DTH was induced as described in Figure 1 legend in wild-type (W.T.) mice and miR-132/212 KO mice and these mice were analyzed for footpad swelling and immunological parameters. **(A)** Showing the percentage of increase in the thickness of each footpad of WT and miR-132KO mice. **(B)** Absolute cell number of CD4+ cells in DLN in various groups. **(C)** qRT-PCR-based expression of mRNA for Foxp3+. **(D)** qRT-PCR-based mRNA expression of IL17. **(E)** qRT-PCR-based mRNA expression of HMGB1. Vertical bars represent Mean ± SEM and significant differences between groups are shown with asterisks (^*^p < 0.05, ^**^p < 0.01, ^***^p < 0.001, ^****^p < 0.0001) based on one-way ANOVA.

To investigate the role of miR-132-3p in the development of DTH in mice upon treatment with TCDD and FICZ, we used wild-type and miR-132/212 Knockout (KO) mice. Upon evaluation of DTH development in these two strains of mice, we noted significant exacerbation of footpad swelling and an increase in draining lymph node cellularity in the mBSA+Vehicle group from miR-132/212 KO mice when compared to the mBSA+Vehicle group from wild-type mice (Figures 8A,B). While TCDD treatment caused a significant decrease in footpad swelling and decrease in DLN cellularity in wild-type mice, TCDD was less effective in reducing DTH and DNL cellularity in miR-132/212 KO mice when compared to wild-type mice (Figures 9A,B). Also, while FICZ when compared to vehicle controls, caused an increase in DTH and DLN cellularity in wildtype mice, it failed to further enhance these indicators in miR-132/212 KO mice when compared to vehicle controls (Figures 9A,B). FoxP3 expression was significantly enhanced in TCDD treated group but not in the FICZ group when compared to the vehicle group from wildtype mice, while TCDD or FICZ when compared to the vehicle group, failed to significantly upregulate FoxP3 in miR-132/212 KO mice (Figure 9C). When we studied IL-17 expression, FICZ but not TCDD was found to upregulate IL-17 in wild-type mice (Figure 9D). However, in miR-132/212 KO mice, both TCDD and FICZ showed increased expression of IL-17. Lastly, when we studied HMGB1 expression in wild-type mice with DTH, TCDD caused a decrease while FICZ caused an increase in the expression of HMGB1. In contrast, in miR-132/212 KO mice, both TCDD and FICZ showed significant upregulation of HMGB1 (Figure 9E). Together, these data suggested that miR-132-3p plays an important role in the development of DTH in mice, and the effect of TCDD and FICZ on mBSA-induced DTH may be regulated, at least in part, through miR-132-3p and HMGB1.

## Discussion

Aryl hydrocarbon receptor (AhR) is an important player in peripheral immune modulation and tolerance. In recent years, there have been several studies demonstrating the role of AhR in the regulation of immune functions (2, 3, 32, 33). A wide range of AhR ligands has been characterized including environmental contaminants, dietary compounds, microbial byproducts, and endogenous mediators (33, 34). Such ligands have been shown to mediate differential effects on immune regulation (2, 3, 34, 35). The most interesting feature of AhR ligands is their ability to induce the differentiation of Tregs vs. Th-17 cells. While some ligands induce Tregs, others have been shown to trigger Th-17 cells (33, 34), and the precise mechanisms remain unclear. In this study, we, therefore, investigated the effect of AhR ligands (TCDD and FICZ) on mBSA-induced DTH in mice and examined the molecular mechanisms involved.

Data from our study showed that TCDD suppressed the development of DTH in wild-type mice by reducing the footpad thickness, lowering DLN cellularity, and reducing infiltration of immune cells into the footpad (Figures 1A–D). Furthermore, there was a significant increase in Foxp3 and TGF-β expression in DLN as well a significant increase in TGF-β concentration in the culture supernatants of DLNs in TCDD-treated groups (Figures 3A–D). Also, TCDD skewed Treg subsets by increasing thymic, peripheral, and Th3 T regulatory cells, while causing no significant effect on Tr1 cells and IL10 expression (Figures 4A–D). FICZ, on other hand, showed opposite effects on the DTH response and T cell differentiation in mice. FICZ exacerbated the DTH response by significantly increasing the footpad thickness, DLN cellularity, and immune cell infiltration into the footpads (Figures 1A–D). FICZ also caused a significant increase in the expression of IL17 and RORγ in DLN. The data obtained from the present study demonstrated that AhR ligands, TCDD, and FICZ, have opposite effects on mBSA-induced DTH in mice even though both TCDD and FICZ have been known to have a high affinity toward AhR. Interestingly, the expression of miRNA in DLN cells revealed that miR-132-3p expression was increased in TCDD treated group while it was decreased in FICZ-treated mice (Figure 6). Thus, when we further investigated the potential targets of miR-132-3p using pathway analysis, we found that miR-132-3p may play a crucial role in regulating the expression of HMGB1, which in turn regulates the expression of FoxP3 that was confirmed by transfection studies as well as the use of miR-132-3p-deficient mice (Figures 7, 8). It should be noted that in the current study, we used flow cytometry to detect the effect of AhR ligands on Tregs (CD4+FoxP3+) and Th17 cells (CD4+IL17+), which was also confirmed by using qRT-PCR assay of the DLN cells. However, because we used the DLN cells for studying other markers such as IL-10, RORγ, TGF-β, and HMGB1, it shows the possible alteration pattern in all cells found in the DLN and not just the CD4+ T cells.

While the ability of TCDD to induce T cells that suppress inflammation was discovered way back in the early 1980s (36), further studies were hindered by the lack of specific markers to detect and characterize such cells. Thus, the discovery of FoxP3 as a master regulator of Tregs (37), enabled this research to progress at a rapid pace. AhR is significantly expressed in both Tregs and Th17 cells (2), whereas, other T cell subsets may not express AhR (34). Although Tregs and Th17 cells are normally reciprocally regulated, AhR ligands have been shown to induce both Tregs and Th-17 cells in varying proportions. For example, TCDD is typically known to induce Tregs whereas FICZ has been shown to trigger Th-17 cells (2, 24, 38). The reason why some ligands trigger Tregs and others Th-17 is unclear but it has been suggested that this may depend on the dose and affinity of the AhR ligand, and the duration of AhR activation (33, 39). Studies from our lab have suggested that this may also be regulated by epigenetic mechanisms induced following activation of AhR (2, 27).

In the current study, therefore, we investigated whether AhR ligands (TCDD and FICZ) treatments would affect miRNA expression in DLN of mice with DTH. Upon analysis of miRNAs profile of the DLN cells of DTH mice treated with Vehicle or TCDD or FICZ, several miRNAs were found to be altered in these groups. However, there was only one miRNA, miR-132-3p, that was differentially expressed in DLN cells treated with TCDD (downregulated) and FICZ (upregulated). AhR activation is well-established to regulate gene expression through the binding of AhR/ARNT complex to specific DNA sequences known as Dioxin Response Elements (DREs). Using *in silico* analysis, we found that miR-132-3p expresses two DRE binding motifs in the promoter region, and thus, further studies are necessary to explore if this pathway constitutes the mechanism through which AhR activation regulates the expression of this miRNA. Upon analysis of miR-132-3p using IPA, we found that miR-132-3p might directly/indirectly be involved in the regulation of Tregs and Th17 cells (Figure 6C). We also observed that miR-132-3p interacts with HMGB1, which was downregulated by TCDD but upregulated by FICZ demonstrating that perhaps downregulated HMGB1 in TCDD caused the induction of Foxp3. Additionally, because Tregs and Th17 cells are reciprocally regulated, induction of Tregs may have inhibited the expression of IL17. On the other hand, HMGB1 was upregulated by FICZ and that might have caused downregulation of Foxp3 expression and upregulated the expression of IL17 leading to suppression of Tregs and an increase in Th17 cells. Previous studies have shown that HMGB1 regulated Treg/IL17 ratio in patients with chronic hepatitis B, in which HMGB1 was significantly higher and induced Th17 cells but suppressed Tregs via TLR4 and IL6 pathway (40). Furthermore, in atherosclerosis, HMGB1 has been shown to modulate Treg/IL17 ratio through inducing apoptosis in Tregs and promoting Th17 cells differentiation (41). Studies are demonstrating the direct and major role the miRNAs play in the regulation of gene expression and the immune responses (42). In recent several studies from our lab, it has been shown that miRNAs played an important role in autoimmunity and inflammation (29, 43–45). These studies showed that miRNAs play a critical role in promoting anti-inflammatory functions and immune suppression (29, 43, 46, 47). Al-Ghezi et al. showed that miR-101b, miR141-3p, and miR142-3p were downregulated in Pertussis Toxin (PTX)-induced inflammation in TCDD-treated mice and showed that TCDD caused induction of Tregs in mice leading to attenuation of PTX-induced inflammatory responses (21). In an earlier study, we found that dietary AhR ligands (indole-3-carbinol [I3C] and 3,3′-diindolylmethane [DIM]) caused suppression of DTH response while FICZ increased the DTH response (27). We found in this study that I3C and DIM increased the expression of miR-132 consistent with the current study showing TCDD being able to induce miR-132. In addition, I3C and DIM also caused a decrease in the expression of miRs such as miR-31, miR-219, and miR-490 that targeted Foxp3, whereas they increased the expression of miR-495 and miR-1192 which targeted IL-17 (27). In contrast, we found that FICZ exerted opposite effects on these miRs (27). The current study corroborates this concept that the ability of AhR ligands to induce Tregs vs. Th17 may be associated with the nature of miRs induced by these ligands. Thus, activation of AhR by certain ligands may alter the expression of miRs which may synergize or antagonize the inflammatory response associated with DTH. For example, TCDD, I3C, and DIM may alter miR-132-3p, miR-31, miR-129, miR-490 leading to FoxP3 induction, while causing significant alterations in the expression of miR-132-3p, miR-495, and miR-1192 to suppress FoxP3 expression while promoting IL-17. In contrast, other ligands such as FICZ may mediate opposite effects on such miRs thereby enhancing the DTH response.

There have been few studies demonstrating the role of miR-132 in inflammation. For example, using transfection studies, it was shown that overexpression of miR-132 enhanced the acetylcholine (Ach)-mediated anti-inflammatory reaction in LPS-treated alveolar macrophages (44). Increased expression of miR-132 decreased the LPS-mediated nuclear translocation of NF-κB and phosphorylation of STAT3 (44). In another study, AhR activation by TCDD was shown to enhance the induction of miR-132 expression and attenuate colitis-associated colon cancer by suppressing the infiltration of macrophages and the production of inflammatory cytokines (45). There are numerous reports on the prominent role played by miR-132 in the nervous system (48–50). For example, miR-132 has been shown to regulate the expression of Nurr1, critical transcription factors involved in the development and differentiation of dopamine neuron development (51).

In summary, in the current study, we demonstrate for the first time that AhR activation during DTH may decrease or enhance the inflammatory response through the expression of HMGB1 via regulation of miR-132. It was interesting to see how two AhR ligands (TCDD and FICZ) reciprocally induced miR-132 which ultimately impacted the induction of Tregs vs. Th17 cells. Our results were supported through the pursuit of both *in vitro* assays (transfection assays using miR-132) and *in vivo* assays (using miR-132/212 knockout mice). Because Th17 cells are involved in the induction of certain autoimmune diseases and Tregs in suppressing such disorders, our studies suggest several key molecules such as miR-132 and HMGB1 as potential targets to treat such inflammatory disorders.

## Data Availability Statement

The raw data supporting the conclusions of this article will be made available by the authors, without undue reservation.

## Ethics Statement

The animal study was reviewed and approved by the University of South Carolina Institutional Animal Care and Use Committee.

## Author Contributions

MN and PN: designed research studies, funding acquisition, resources, and supervision. OA: performed all the experiments under supervision of PN and MN. OA data acquisition, data analysis, writing, and original manuscript draft. OA, WN, and MS contributed to analyze data. NS, SC, MN, and PN: writing, editing, and revisions. All authors contributed to the article and approved the submitted version.

## Conflict of Interest

The authors declare that the research was conducted in the absence of any commercial or financial relationships that could be construed as a potential conflict of interest.

## Abbreviations

AhR: Aryl hydrocarbon receptor
ARNT: aryl hydrocarbon receptor nuclear translocator
DIM, 3: 3′-diindolylmethane
DLN: draining lymph node
DREs: dioxin response elements
DTH: delayed-type hypersensitivity
EAE: Experimental Autoimmune Encephalomyelitis
ELISA: enzyme-linked immunosorbent assay
FICZ, 6-formylindolo [3: 2-b] carbazole
Foxp3: Forkhead box P3
HMGB1: High Mobility Group Box 1
I3C: indole-3-carbinol
IL6: Interleukin 6
IL17: Interleukin 17A
IPA: Ingenuity Pathway Analysis
mBSA: methylated Bovine Serum Albumin
miRNA: MicroRNA
PTX: pertussis toxin
RORγ: retinoic acid-related orphan receptor γ
RT-qPCR: reverse transcription-polymerase chain reaction
TCDD: 2, 3, 7, 8-Tetrachlorodibenzo-p-dioxin
TGF-β: Transforming growth factor-beta
TLR4: Toll-like receptor 4
Tregs: regulatory T cells
XREs: xenobiotic response elements.
